# Physical Fitness and Inflammatory Response to the Training Load of Wheelchair Rugby Players

**DOI:** 10.3390/ijerph19042228

**Published:** 2022-02-16

**Authors:** Łukasz Szymczak, Tomasz Podgórski, Jacek Lewandowski, Arkadiusz Janiak, Edyta Michalak, Katarzyna Domaszewska

**Affiliations:** 1 Faculty of Health Sciences, Calisia University, 62-800 Kalisz, Poland; l.szymczak@akademiakaliska.edu.pl (Ł.S.); lewandowski@awf.poznan.pl (J.L.); arjan7@wp.pl (A.J.); 2 Department of Physiology and Biochemistry, Poznan University of Physical Education, 61-871 Poznan, Poland; podgorski@awf.poznan.pl (T.P.); michalak@awf.poznan.pl (E.M.); 3 Department of Musculoskeletal Physiotherapy, Poznan University of Physical Education, 61-871 Poznan, Poland

**Keywords:** spinal cord injury, testosterone, cortisol, creatinine, overtraining

## Abstract

The aim of the study was the evaluation of the hormonal response of wheelchair rugby participants under the half-year training cycle. The study sample included 11 members of the Polish national wheelchair rugby team with spinal cord injury at the cervical level, ranging in age from 21 to 41 years, body weight (72.2 ± 11.53 kg), and body height (182.3 ± 6.11 cm). The disabled individuals with spinal cord injury subjected to the study constitute a homogeneous group in terms of age, body height, weight, and injury level. The study was carried out at the beginning and at the end of a 6-month training period. In the first and second examination, measurements of the peak oxygen uptake (peakVO_2_) and blood biochemical analysis were performed (Lactate dehydrogenase (LDH) activity and concentration of creatinine (Cr), total testosterone (TT), free testosterone (FT), and cortisol (C)). A significant change was observed in the concentration of C in the Wheelchair Rugby players’ blood between two research periods (*p* < 0.05 (ES:0.76)) and a correlation between the post-training change in FT/C concentration and the change in Cr concentration (r = −0.6014, *p* < 0.05). The 6-month training period did not result in overloads within the group of players. However, due to the significant loss of the capacity of the spinal cord injury (SCI) and the possibility of a life-threatening trend, the anabolic/catabolic status of the players should be monitored using blood biochemical indices.

## 1. Introduction

Spinal cord injury causes impairment, or total loss of the motor and sensory functions in the areas of thorax, upper and lower limbs, depending on the severity of the damage [1]. The vast majority of such injuries stem from traffic accidents, bullet wounds, falls, jumps into the water, and sports injuries. For the elderly, the injury factors may be a disc, primary or metastatic neoplastic lesions, developing inflammatory processes in the area of the damage, or compression by abnormal blood vessels. The most common injuries occur in the cervical region or at the border of the thoracolumbar region. However, almost half are injuries between C4 and C7 [2,3]. Impairment of the skeletal muscle function, as well as a physiological adaptation to the physical effort, impacts everyday life activities. The rehabilitation process of such persons, apart from health and compensation components, aims at the mobilization of the patient to function together with healthy persons. Accounting for modern knowledge, it was the right decision to introduce sportsmanship to the rehabilitation programs of patients with SCI [4]. SCI person activation practices may apply to several sports disciplines, including wheelchair basketball, tennis, or boccia. One of the sports dedicated to persons with SCI damage is wheelchair rugby [5,6]. This sport, despite the emergence of persons with other impairments, is still dominated by persons with spinal cord injuries involving the cervical spine (tetraplegia), which is one of the most severe musculoskeletal disabilities. Similar to other Paralympics disciplines, the problem of injuries and overloads of the musculoskeletal system in wheelchair rugby is not widely and thoroughly researched. When identifying damages and overloads in handicapped sports, it is important not only to account for the sport’s characteristics but also for the type of disability of the participants. Injuries that occur during wheelchair rugby training and matches include those associated with falls and wheelchair impacts (wheelchair rugby is a contact sport and such incidents are acceptable) [5].

Another aspect of damages and overloads is the everyday life in which most of the participants use wheelchairs. Sports-related damages and overloads can overlap with overloads from daily wheelchair use.

Martin et al., Burnham et al., and Castro et al. in their research on the persons with SCI observed the transformation of slow-twitch fibers into fast-twitch fibers, primarily glycolytic [7,8,9]. Muscle glycogen stores and muscle aerobic potential are reduced due to decreased mitochondria and muscle fiber transformation. The abolition of sympathetic nervous system function is responsible for the rapid development of muscle fatigue in a person with cervical SCI. Incorrect physiological conditions sympathetic innervation is responsible for increasing blood flow to working skeletal muscles, therefore intensifying the cell oxygen supply, and non-muscular energy substrates such as glucose and free fatty acids. Reduced muscle blood flow in an injured situation, lack of muscle pump function, impaired cardiovascular reflexes lead to metabolic changes in the muscle and accumulation of lactate. Increased glycolytic metabolism, with concomitant disturbance of homeostasis, leads to the rapid development of fatigue in working muscles [8,10].

The disturbance in maintaining proportions between the time of training and biological rejuvenation results in decreased physical capability of the participant, greater risk of injury, and decreased body immunity, which is particularly dangerous for this group of patients during the increase in COVID-19 (SARS-CoV-2) infections [11,12,13]. Hence, the monitoring of fatigue levels using physiological or biochemical indicators will allow for sustaining the participants’ optimal health conditions and life comfort despite numerous physical conditions affecting them.

Biochemical indicators of muscle breakdown or cell membrane damage include measurement of creatinine (Cr) or creatine kinase (CK; EC 2.7.3.2) lactate dehydrogenase (LDH; EC 1.1.1.27). Most often the testosterone (T) and cortisol concentrations in the blood are used for the diagnosis of the overload state. The literature analysis indicates that the development of anabolic–catabolic balance is not only influenced by the training’s intensity and duration, but also by the training volume, or atmospheric conditions [14,15]. Cortisol is considered to be a catabolic hormone because it stimulates glycogenolysis in the liver by increasing the glucose concentration in blood. It inhibits muscle uptake of amino acids and limits muscle protein synthesis [15,16]. Testosterone via its anabolic impact on the body causes the increase in the synthesis of muscle tissue, forming mass and muscle strength. At rest, only 1% of testosterone circulates in the blood in the free form (FT) and is metabolically active. The remaining part is linked to the sex hormone-binding globulin (SHBG) and with albumins [16,17]. Analysis of the testosterone/cortisol T/C ratio is not only a good indicator of the body’s metabolic balance but is also found in the literature to be an indicator of peripheral and central fatigue in athletes [18,19,20]. Low values of the TT/C and FT/C indicators are proposed to be early biochemical indicators of exercise-induced fatigue [14]. Esspecially in extreme cases, intensified catabolic and limited anabolic processes during athletes’ training cycle, TT/C and FT/C ratios seem to be appropriate indicators of the overreaching or overtraining syndrome [20]. The value of these indicators was noticed, for example, in non-disabled rugby players [21]. For athletes with quadriplegia and limited active muscle mass, these conditions can occur at even low exercise loads, and the effects of overtraining are far more dangerous to the athlete’s health and even life. In this group of persons the katabolic changes increase in muscle even in resting conditions. There is evidence that the level of endogenic, anabolic hormones may be decreased. Decreasing the testosterone and growth hormones concentration and through them insulin-like growth factor-1 (IGF-1) may intensify unbeneficial body mass composition changes, decreasing the fatigue tolerance [22,23,24]. Wang et al. indicated in their research that in the group of 63 patients with spinal cord injury, a comparable number of individuals had both decreased and increased blood testosterone concentrations at rest [25]. Hence, the analysis of the concentration of selected hormone levels in the group of elite wheelchair rugby competitors in different stages of preparatory training for the important sporting event was proposed, which will allow for the evaluation of the direction and character of changes.

The aim of the study was the analysis of the hormonal response (TT, FT, C, and TT/C, FT/C ratio) of wheelchair rugby participants under the training.

## 2. Materials and Methods

The study protocol was approved by the Ethics Committee for Human Research of the Poznan University of Medical Sciences (Ethics Approval Number: 405/12). The study was conducted according to the Declaration of Helsinki and the National Statement. All participants in this study gave their written informed consent.

The study sample included 11 members of the Polish national wheelchair rugby team with spinal cord injury at the cervical level, ranging in age from 21 to 41 years, body weight (72.2 ± 11.53 kg), body height (182.3 ± 6.11 cm). The disabled with spinal cord injury subjected to the study constitute a homogeneous group in terms of age, body height, weight, and the injury level. All of them were tetraplegics with cervical spinal cord injury at levels C5–C7. Wheelchair rugby classification system, used to assess the subjects came from the ISMGF/ISMWSF (International Stoke Mandeville Games Federation/International Stoke Mandeville Wheelchair Sport Federation) and American Spinal Injury Association (ASIA) Impairment Scale, medical classification system (Table 1).

Training period between research dates was characterized by build-up of general capability, with a particular focus on the aerobic capacity and improving elements of game tactics. In the later period, the participants focused their training on the speed. Research in the 2nd term took place after the beginning in a target competition, near the immediate start.

### 2.1. Assessment of Aerobic Fitness (Graded Exercise Test Protocol)

The subjects performed a cardiopulmonary exercise test with increasing intensity on the Sweedish MONARK REHAB TRAINER 881E manual ergometer, manufactured by Monark Exercise AB (Vansbro, Sweden) and specially adjusted to functional abilities of research participants. The initial workload amounted to 10 Watts was successively increased by 10 Watts every 3 min until the subjects achieved maximum individual workloads or refused to continue the effort. Peak oxygen uptake (peakVO_2_) was measured in absolute and relative values with a German Jeager Oxycon Mobile gas analyser (Viasys Healthcare, Höchberg, Germany) [26].

### 2.2. Methodology of Biochemical Marking

Blood samples were collected before exercise tests (two days after the last intensive training unit) to exclude the influence of training loads on biochemical variables tested. Blood samples were taken from the ulnar vein using a S-Monovette syringe tube (Sarstedt, Nümbrecht, Germany) then placed in tubes containing a clot activator and centrifuged (1500 g, 4 °C, 4 min) in order to separate the serum. The samples were frozen and stored at −75 °C until the time the analyses were performed. Lactate dehydrogenase (LDH) activity and concentration of creatinine (Cr) was determined with the use of the Accent 200S (Cormay, Poland) biochemical analyser and sets of enzymatic reagents (Cormay, Poland). The sensitivity of the sets was 6.6 U/L for LDH and creatinine 7.07 (micro) mol/L (intra-assay coefficient of variation [CV], <10%). Concentrations of total testosterone (TT), free testosterone (FT), cortisol (C), were measured using the ELISA immune-enzymatic method (AssayPro LLC, St. Charles, MO). The sensitivity of the ELISA kits was as follows: 0.083 ng/mL ([CV], 3.61%), 0.018 pg/mL ([CV], 5.96%), 2.5 ng/mL ([CV], 5.63%).

The samples were read using a Synergy 2 SIAFRT multi-detection microplate reader (BioTek, Winusky, VT, USA) at the manufacturer’s recommended wavelength.

### 2.3. Training Program

The preparation cycle of the Polish Wheelchair Rugby Team in 2012 included 30 weeks of training (microcycles) in preparation for the start in the target event—the International Metro Cup Tournament.

- The first stage of preparations, covering the period from March to mid-May 2012 (microcycles 1–12), was focused on building aerobic capacity (raising the level of fitness of players), strengthening muscle mass and perfecting tactical elements of the game (raising the tactical level of players, shaping the reaction speed to changing tactics of the opponent during the game and gathering players from different sports clubs together).
- The second stage of preparations, covering the period from mid-May to the end of August 2012 (microcycles 13–26), was focused on maintaining aerobic capacity and strength endurance (the beginning of the stage) and speed endurance (the end of the stage), improving elements of game tactics (shaping the habits of observation and analysis during physical effort, learning team attitudes).
- The third stage of preparation, covering the period of September 2012 (micro-cycles 27–30), (direct starting preparation) is shaping the speed abilities of players and improving elements of game tactics (evaluation of observation and analysis habits during physical effort).
- Stage IV, covering September/October 2012 (microcycles 31–32) of the year, was the start of the target event.
- The fifth stage, covering the period from October to November 2012 (microcycles 33–36), was a transitional period (retrenchment).

The exact training plan realised by the players during the period covered by the research is shown in Table 2.

### 2.4. Statistical Analysis

All data are presented as mean, standard deviation (SD). The normality of distribution was tested with the Shapiro–Wilk test. The differences between paired and normally distributed variables were analysed using the Wilcoxon test. Spearman’s rank analysis was used to calculate correlation coefficients. The level of statistical significance was set at *p* ≤ 0.05. The obtained results were analysed statistically using the Dell Inc. (2016) Dell Statistica 13 software (Tulsa, Oklahoma, USA). Effect sizes (ES) were calculated as the difference between means divided by the pooled standard deviation using Cohen’s criteria; effect sizes <0.20 and <0.50 were considered small, <0.50 and <0.80 medium, and <0.80 large [27].

## 3. Results

Resting testosterone levels in the blood of the FT and TT fractions were within the physiological limits of this parameter. The blood cortisol concentration was similar. A slight increase in LDH activity and a decrease in creatinine concentration were found. Values below the lower limit of the reference values were found in 9 persons (82%) in the first research period and 8 persons (73%) in the second research period.

Our research showed a significant change in the concentration of cortisol in the Wheelchair Rugby players’ blood between two research period. (*p* < 0.05 (ES:0.76)) and a correlation between the post-training change in FT/C concentration and the change in creatinine concentration (r = −0.6014, *p* < 0.05) There was also a post-training insignificant decrease in peakVO_2_ (*p* > 0.05), resulting from the increase in body weight of the subjects in the second term of the study (Table 3).

## 4. Discussion

The size of exercise loads during training camps varies and is dependent on the preparatory period of participants. It needs to be stated that weekend training camp consists of 3 training units lasting 3–4 training hours, and of one theoretical training unit. In the 1st preparatory stage during the weekend camps, participants cover the biggest number of training kilometers in comparison with the speed exercises. This ratio changes with the realization of subsequent training macrocycles.

In our research, the intensive 6-month-long training impacted the selected metabolic parameters on the verification of the overextension state occurrence. Such a state in healthy sports-playing participants requires the application of effective biological regeneration treatments, and decrease of several weeks in the training severity. Healthy persons with high levels of physical capacity have developed effective mechanisms of post-exercise rejuvenation; however, among persons with SCI this mechanism is severely limited. The typical over-exercise symptoms are the loss of physical capacity, loss of muscle mass, the occurrence of the negative nitrogen balance. Such changes are visible in the nervous system functioning, and changes to the secretion of the metabolic hormone [28,29].

From the point of view of training, there are many similarities in the physical reactions to the over-training of healthy and handicapped participants. The hormonal response to the applied workload may differ. Individuals with acquired disabilities respond differently to training loads than individuals with disabilities from birth. It has been demonstrated during the observational studies that persons with acquired disabilities are more motivated to push through their limitations and have stronger stress reactions to the training stimulus, therefore they may react with greater cortisol secretion [6].

The amount of the training load of the rugby wheelchair players during training camps varies and is dependent on the preparation period of the participants. It ought to be noted that the weekend training camps consist of three practical training units of 3–4 h each, and one theoretical training unit. In the first stage of preparation during the weekend training camps the participants cover the greatest amount of training kilometers in comparison with speed training. The proportion changes with the realization of the training macro-cycles. The first date of participants’ testing was at the beginning of the preparatory period, during which the general fitness of players was built, especially aerobic capacity. The second date of testing was on the period immediately after the start of the target event. In this period, it was expected to record a significant overload of the body, and damage to the muscular system was expected.

In our research, we concluded that participants with tetraplegia had normal resting testosterone and cortisol blood levels (Table 3). Castellani et al. found a statistically significant (*p* < 0.05) decrease in this hormone in the wheelchair participants in comparison with healthy ones. They found a greater increase in exercise-induced testosterone concentration in wheelchair participants in comparison with healthy ones. They also showed a higher exercise-induced increase in testosterone concentrations in subjects with disabilities. However, there were no differences in cortisol concentrations at rest as well as after exercise [30]. The physical mechanisms leading to an increase in exercise-induced testosterone levels are a result of both its production, breakdown, and excretion. Exercise-induced changes in plasma volume can also modulate its concentration in the blood. In the research of Wheeler et al., it can be concluded that some participants diagnosed with SCI deliberately increase the catecholamine (CA) output by triggering the autonomic dysreflexia mechanism to increase their performance. Therefore, it is assumed that high exercise blood testosterone concentration is a response to catecholamine output or a protective response induced by cortisol [31,32].

There is little research on the biochemical reaction of wheelchair participants to the training load. Limiting the search to the rugby participants gives very few publications useful for the critical analysis of own research results. In the research of Bizzarini et al. on the impact of a 6-month training regime on the persons diagnosed with SCI (6 persons with damaged cervical region, 15 with the damaged thoracic and lumbar regions). There were no significant changes to the testosterone and cortisol levels after the training period. General ratio TT/C, currently perceived to be the indicator of overtraining, did not decrease in the presented study. The authors explain this fact by the lack of intensification of catabolic metabolism in response to physical activity or by the limited ability of the body to metabolically adapt to the intensity of physical exercise [33].

In the research on healthy participants, Ahtiainen et al. described post-training decrease in total testosterone levels, as well as the ratio of TT/C caused by too high training loads [34]. In our research on the SCI participants, the increase in the above-mentioned markers was not recorded, which may be indicative of the high physical capability of the research participants as well as the training load appropriate for their health condition capabilities. This is also indicated by the lack of a post-workout decrease in the peakVO_2_, which determines an athlete’s oxidative capacity level. The importance of cortisol analysis in the diagnosis of overload conditions in athletes was mentioned by Michailidis in his study on football players [35]. Cortisol is the stress hormone secreted both as a result of physical exertion factors as well as stress factors connected to physical activity. In many research, the post-training increase is dependent on the exercise intensity and acidification levels [36,37,38]. Based on the Michailidis results it can be concluded that the lack of changes in the C concentration during the participant’s preparation cycle is indicative of their good psychophysical preparation [35]. Barboza et al. indicate that athletes with neurological damage (SCI), unlike other disabilities (poliomyelitis, amputation), do not respond to saliva secretion of cortisol in basketball players in wheelchairs [39]. Eventually this can cause problems with the interpretation of the SCI athletes’ body response to the effects of training intensity.

Lack of changes in LDH activity or creatinine concentration in our research indicates the sustained muscle cells stability and no damage to them as a result of the training regime. The increase in cortisol in the second term of research may be reflective of stress caused by the matches itself during the target event in the year of the research. A slight increase in LDH enzyme activity and a decrease in creatinine concentration; values below the lower limit of reference values were found in more than 80% of the subjects, most likely due to increased creatinine excretion in urine [40]. Kaji et al. based on the conducted research proved the decrease in urinary creatinine excretion in a group of patients with SCI based on their study. The factor limiting the precision of defining the accuracy of the renal clearance rate in our study was lack of the creatinine concentration in urine measurements [40]. The long-term research of Elmelund et al. on the creatinine levels in the blood of persons with SCI prove that the decrease in this metabolite is caused by the loss of muscle mass with age, which naturally occurs in healthy persons as well. In persons with SCI, this mechanism is accelerated due to the extensive muscle mass loss due to injury itself [41].

The lack of changes in the creatinine blood concentration in blood in participants studied in the second term indicates sustaining the stable muscle mass and lack of damage to the kidneys. We are more interested in the presented findings in terms of the occurrence of possible overload changes resulting in a decrease in muscle mass in individuals with symptoms of overtraining of a peripheral nature. Particularly since, in the case of athletes with SCI, the source of overload is a matter of everyday life, in which most athletes use wheelchairs.

Monitoring of the metabolic hormonal state (TT/C) will allow for both trainers and psychologists treating handicapped patients to fully control the degree of physical participant’s preparation as well as their psychological state and motivation. It may also serve as a sensitive biochemical marker of the way to deal with physical limitations accounting for the SCL type, functional classification, or disability due to impairment (acquired or congenital) [42,43].

## 5. Conclusions

The 6-month training period did not overstrain the group of players. However, due to the significant loss of SCI capacity and the possibility of a life-threatening trend, the anabolic/catabolic status could be used to diagnose early states of overload.

### Limitation of the Study

The study was conducted only on persons physically active with spinal cord injuries. In order to obtain a complete picture of anabolic/catabolic biochemical changes under the influence of a 6-month training period, tests would have to be carried out on physically inactive men with spinal cord injury. Unfortunately, this study did not obtain the right consent to search for this group of people.

## Figures and Tables

**Table 1:** Classification points, spinal cord injury level, American Spinal Injury Association (ASIA) Impairment Scale of the subjects with spinal cord injury [11].

No	Spinal Cord Injury Level	American Spinal Injury Association (ASIA) Impairment Scale	Classification Points
1	C6–C7	A	2
2	C5–C6	A	2
3	C6	A	2
4	C6–C7	A	2
5	C6–C7	A	2.5
6	C5–C6	A	2
7	C6–C7	A	2
8	C6–C7	A	2
9	C5	A	2
10	C5–C6	A	1
11	C6–C7	A	0.5

**Table 2:** Wheelchair rugby players’ weekly training frequency in a macrocycle (training load description).

Training Goal	Week	Type of Training	Type of Activity	Duration [min]	Intensity [% max]	No. of Training Sessions per Week
Aerobic endurance (development and maintenance)	1–12	Constant intensity aerobic training	Wheelchair push (indoor, outdoor)	60–100	50–60	3
	13–32	Constant and variable intensity aerobic training	Wheelchair push (indoor)	60–100	60–70	2
Speed endurance (anaerobic)	27–32	Interval training	Wheelchair push (indoor)	15–20	70–90	1
Development of muscle strength	1–12	Strength training	Gym	60–80	60–70	2
Maximum strength	13–26	Strength training/functional training	Gym/fitness	40–80	80–90	1
Strength endurance	13–32	Circuit training	Gym/fitness	40–80	50–60	1
Power/Capacity	13–32	Speed and strength training	Gym/fitness	40–80	60–70	1
Stabilisation (deep muscle training)	1–26	Circuit training (repetition)	Fitness room	30	-	1
Tactics/Team play	1–12	Small tactics training	Wheelchair rugby (key, half court play)	120–150	50–75	3
	13–32	Training of small and large tactics	Wheelchair rugby (key, half court play, regular games)	120–150	50–90	3

**Table 3:** Basic characteristics (x ± SD) of physiological and biochemical parameters measured in first and the second research period.

Parameter	First Research Period	Second Research Period	*p*-Value
Cr [µMol/L]	57.1 ± 13.53	57.7 ± 12.14	1.0000
LDH [U/L]	310.7 ± 68.09	307.2 ± 49.02	0.8139
TT [ng/mL]	4.2 ± 0.90	4.2 ± 1.53	0.8753
TF [pg/mL]	8.2 ± 2.71	8.2 ± 3.69	0.8139
C [ng/mL]	150.7 ± 60.14	207.1 ± 52.61	0.0342 *
TT/C	0.03 ± 0.01	0.02 ± 0.01	0.0843
TF/C	0.06 ± 0.03	0.04 ± 0.02	0.0994
Body mass [kg]	72.2 ± 11.53	73.5 ± 7.69	0.5939
peakVO_2_ [mL/kg/min]	16.8 ± 3,49	15.9 ± 3.52	0.1386

Data are presented as mean ± SD, Cr = creatinine, LDH = Lactate dehydrogenase, TT = total testosterone, FT = free testosterone, C = cortisol, peakVO_2_ = peak oxygen uptake. * *p* < 0.05

## Data Availability

The data presented in this study are available on request from the corresponding author. The data are not publicly available due to the consent provided by participants on the use of confidential data.
